# Inflammatory and Immune Microenvironment in Myeloproliferative Neoplasms: Pathogenic Mechanisms and Therapeutic Opportunities

**DOI:** 10.3390/cancers18162718

**Published:** 2026-08-21

**Authors:** Faride Kaikavoosnejad, Ali Keyhani, Seyyede Sepide Ashraf Moosavi, Milad Verdi, Mohammad Sepehr Yazdani, Khadijeh Dizaji Asl, Zeinab Mazloumi, Hamed Mirzaei, Ali Rafat, Reza Nejati

**Affiliations:** 1 Student Research Committee, Kashan University of Medical Sciences, Kashan, Iran, 8715988141; keikavoosnejad-f@kaums.ac.ir (F.K.); keyhani-ali@kaums.ac.ir (A.K.); ashrafmousavi-s@kaums.ac.ir (S.S.A.M.); mr.msyazdani@gmail.com (M.S.Y.); 2 Department of Histopathology and Anatomy, TaMS.C., Islamic Azad University, Tabriz, Iran, 5157944533; kh.dizaji@iau.ac.ir; 3 Department of Medical Applied Cell Sciences, Faculty of Advanced Medical Sciences, Tabriz University of Medical Sciences, Tabriz, Iran, 5166614711; mazloomi@tbzmed.ac.ir; 4 Research Center for Biochemistry and Nutrition in Metabolic Diseases, Kashan University of Medical Sciences, Kashan, Iran, 8715988141; mirzaei-h@kaums.ac.ir; 5 Anatomical Sciences Research Center, Institute for Basic Sciences, Kashan University of Medical Sciences, Kashan, Iran, 8715988141; 6 Department of Pathology, Fox Chase Cancer Center, Temple University Health System, Philadelphia, PA 19111, USA

**Keywords:** myeloproliferative neoplasms (MPNs), chronic inflammation, immune dysregulation, JAK–STAT pathway, tumor microenvironment (TME)

## Abstract

Philadelphia-negative (Ph-negative) myeloproliferative neoplasms (MPNs) include polycythemia vera (PV), essential thrombocythemia (ET), and primary myelofibrosis (PMF), which are clonal hematopoietic disorders caused by somatic gene mutations in the JAK2, CALR, or MPL genes. Mutations activate the JAK–STAT pathway and disrupt NF-κB signaling, leading to a chronic inflammatory state caused by pro-inflammatory cytokines and reactive oxygen species (ROS). This altered microenvironment causes serious clinical features of the disease, such as bone marrow fibrosis, splenomegaly, vascular niche remodeling, and a greater probability of thrombosis or secondary leukemic transformation. Concurrently, MPNs cause both severe immune dysregulation and tumor evasion, as evidenced by progressive lymphopenia, T and B cell exhaustion, Natural Killer cell maturation arrest, and the accumulation of myeloid-derived suppressor cells. Although FDA-approved JAK1/JAK2 inhibitors ruxolitinib, fedratinib pacritinib and momelotinib effectively reduce splenomegaly and symptom burden and have demonstrated survival benefits in clinical trials, their ability to eliminate malignant clones or induce durable disease modification remains limited, and disease progression continues to occur in most patients. Finally, this review assesses the complex immunological dysfunction and chronic inflammatory dysregulation that characterize Ph-negative MPNs, as well as emerging therapeutic strategies, emphasizing the importance of fully understanding these intricate microenvironmental mechanisms for the identification and development of novel precision treatment targets.

## Simple Summary

Philadelphia-negative myeloproliferative neoplasms (Ph-negative MPNs), including polycythemia vera, essential thrombocythemia, and primary myelofibrosis, are blood cancers driven mainly by mutations in the JAK2, CALR, or MPL genes. These mutations alter cellular signaling and promote persistent inflammation through increased production of inflammatory molecules and reactive oxygen species. This inflammatory environment contributes to bone marrow fibrosis, splenomegaly, vascular changes, thrombosis, and, in some patients, progression to acute leukemia. MPNs also disrupt the immune system, leading to impaired T- and B-cell function, abnormal Natural Killer cell maturation, and accumulation of immunosuppressive myeloid-derived suppressor cells. Although JAK1/JAK2 inhibitors can reduce symptoms and spleen enlargement and may improve survival, they generally do not eliminate malignant cells or prevent disease progression. Current treatment strategies therefore remain focused largely on controlling disease manifestations while emerging therapeutic approaches aim to target the inflammatory and immune mechanisms that contribute to disease progression.

## 1. Introduction

Philadelphia-negative myeloproliferative neoplasms (Ph-negative MPNs) are a group of diseases defined by aberrant proliferation of myeloid cells in the peripheral blood [1]. Polycythemia vera (PV), primary myelofibrosis (PMF), and essential thrombocythemia (ET) are the three most common BCR-ABL-negative MPNs [2]. The incidences of ET and PV are comparable, an incidence of 1–2 per 100,000 person-years in the US, although PMF is less prevalent, having an incidence of 0.3 per 100,000 person-years [3]. While some historical and current classifications include chronic myeloid leukemia (CML) under the broad umbrella of myeloproliferative disorders due to shared features of myeloid hyperproliferation, CML is pathognomonically defined by the presence of the Philadelphia chromosome (BCR-ABL1 fusion) and exhibits a distinct pathogenesis, clinical course, and therapeutic response to tyrosine kinase inhibitors. Therefore, CML is conventionally considered a separate entity from the classical Philadelphia-negative MPNs, which comprise PV, ET, and PMF [4]. This review focuses exclusively on these Philadelphia-negative (BCR-ABL-negative) MPNs, consistent with the World Health Organization (WHO) classification system that distinguishes CML from classical MPNs based on genetic and clinical criteria [5].

Clinical characteristics of ET, PV, and PMF include thrombocytosis, erythrocytosis and leukocytosis, and bone marrow fibrosis often accompanied by progressive splenomegaly, cytopenias, and constitutional symptoms, respectively [6]. MPNs differ in their prognoses, whereas myelofibrosis (MF) often reduces life expectancy significantly compared to PV or ET [7]. A subset of MPN patients may progress to a blast phase resembling acute myeloid leukemia (secondary AML), which is frequently resistant to conventional therapy. The risk of leukemic transformation varies by subtype: approximately 1.5% of ET cases, 4–7% of PV cases, and 11–20% of PMF cases may progress to secondary AML. This transformation is influenced by age, prior chemotherapy exposure, and the presence of specific genetic alterations [8].

Additionally, thrombotic and bleeding episodes are a characteristic that complicates the clinical progression of MPNs [9]. Consequently, relative to the healthy population, MPN cases worldwide have a reduced life expectancy and a lower quality of life [10].

Myeloid progenitor cell clonal proliferation and single or multilineage hyperplasia result from somatic mutations that constitutively activate physiologic signaling pathways in hematopoietic stem cells and progenitor cells [8]. Despite differences between them, MPNs have important clinical characteristics in common, including comparable bone marrow architecture, an increased risk for venous and arterial thrombosis, and a chance of developing acute leukemia or secondary MF. These phenotypic shared characteristics were recognized long before the discovery of activating Janus kinase 2, Calreticulin (CALR), Myeloproliferative leukemia (MPL), and the discovery of aberrant Janus Kinase-Signal Transducer and Activator of Transcription (JAK–STAT) signaling, which has since improved our understanding of these diseases [4]. Approximately 95% of PV patients have the JAK2V617F mutation, and almost all other cases have mutations within JAK2 exon 12 [11]. The JAK2V617F mutation is present in about 50% of ET and PMF patients, with CALR or MPL (the gene encoding the thrombopoietin receptor) mutations dominating in the remaining cases [4]. These clonal markers have been incorporated into the WHO diagnostic criteria for PMF (58% JAK2, 25% CALR, and 7% MPL mutation frequency), ET (60% JAK2, 22% CALR, and 3% MPL), and PV (98% JAK2); approximately 10–15% of those diagnosed with PMF or ET do not have all three driver mutations, and they are commonly referred to as “triple negative” [12].

Growing evidence suggests that inflammation plays a critical role in promoting MPN formation and influencing disease progression in conjunction with recent breakthroughs in molecular characterization [4,8,12].

Furthermore, a number of studies indicated that MPNs have severe immune system disruption, which promotes tumor escape mechanisms [13]. Following acquisition of a major driver mutation, including JAK2V617F, CALR, as well as MPL, a multifaceted paracrine network of inflammatory growth factors and cytokines exerts a significant effect on both the clinical manifestation and clonal progression associated with MPNs [4].

Many inflammatory signaling molecules activate the JAK–STAT pathway, thereby increasing the viability, proliferation, and survival of numerous cell types, including malignant tumor cell clones [14,15,16]. However, pro-inflammatory signaling is enhanced in neutrophils, monocytes, and endothelial cells in the tumor microenvironment (TME) in addition to premalignant and malignant MPN cell clones, thereby contributing substantially to tumor immune evasion. Activation of JAK2 signaling pathways increases the generation of inflammatory cytokines by activating NF-κB signaling [8].

An increasing number of studies have established that MPNs are frequently linked to a pro-inflammatory cytokine milieu. These changes are accompanied by expansion of the macrophage/monocyte compartment and myeloid-derived suppressor cells, alongside functional alterations in T cells, dendritic cells, and natural killer (NK) cells [12]. Pro-inflammatory chemokines and cytokines, such as TNF-α, IL-2, IFN-α, and IL-23, promote aberrant T cell polarization and promote a pathogenic phenotype of Th17 cells. Notably, exhausted PD-1+ T cells predominate in lymphocyte subset analysis, indicating deficient immune surveillance. At the same time, chronic inflammation as well as stromal activation result from changes in neutrophil apoptosis [4]. Bone marrow remodeling and megakaryocyte enlargement are mediated by fibrosis-promoting factors like TGF-β and IL-13, whilst vascular niche changes, especially in PV, are enhanced by VEGF and other angiogenic factors [4].

Although this abnormal overproduction of functionally mature cells may at first appear as an indolent disorder, with patients, particularly those with ET or PV, exhibiting only asymptomatic peripheral cytoses; however, all MPNs have the potential to progress to end-stage MF with bone marrow (BM) failure or overt acute leukemia [17]. Transformation of MPNs to secondary AML is observed in roughly 1.5% of ET cases, 4–7% of PV cases, and 11–20% of PMF cases [8]. The mechanisms underlying leukemic transition are still not well understood, although it has been demonstrated that the risk of developing secondary AML is raised by other factors, such as age and prior chemotherapy exposure [8].

This review distinguishes itself from recent narrative reviews on MPN immunology through three key contributions. First, we provide an integrated framework that connects driver mutations (JAK2, CALR, MPL), inflammatory signaling pathways, and immune cell dysfunction across all three classical Ph-negative MPN subtypes PV, ET, and PMF emphasizing how mutation-specific immune profiles influence disease phenotype and clinical progression. Second, we present a comprehensive analysis of the bidirectional relationship between chronic inflammation and bone marrow niche remodeling, incorporating emerging evidence on the neuro-hematopoietic axis, osteoblast-osteoclast imbalance, and vascular niche alterations that have not been systematically integrated in previous reviews. Third, we critically evaluate the therapeutic landscape beyond JAK inhibition, with particular emphasis on CALR-directed immunotherapies (monoclonal antibodies, bispecific T-cell redirecting antibodies, peptide vaccines), epigenetic modulators (BET, LSD1, HDAC inhibitors), and combination strategies that are currently in clinical development. While recent reviews have addressed cytokine landscapes or immune dysregulation in isolation, our synthesis of these interconnected mechanisms provides a clinically oriented framework for understanding disease pathogenesis and identifying novel precision treatment targets.

### Search Strategy and Selection Criteria

This narrative review was conducted by systematically searching the PubMed, Scopus, and Web of Science databases for English-language articles published between January 2000 and March 2026. The search strategy employed the following keywords and Boolean operators: (“myeloproliferative neoplasms” OR “MPN” OR “polycythemia vera” OR “essential thrombocythemia” OR “primary myelofibrosis” OR “myelofibrosis”) AND (“inflammation” OR “inflammatory” OR “cytokine” OR “immune” OR “immunology” OR “immune evasion” OR “immune surveillance” OR “T cell” OR “NK cell” OR “myeloid-derived suppressor cells” OR “macrophage” OR “neutrophil”) AND (“JAK2” OR “CALR” OR “MPL” OR “JAK–STAT” OR “NF-κB”) AND (“therapy” OR “treatment” OR “JAK inhibitor” OR “immunotherapy” OR “vaccine” OR “combination therapy”). Additional articles were identified through manual screening of reference lists from retrieved reviews and original research articles. Priority was given to peer-reviewed original research, clinical trials, systematic reviews, and meta-analyses. Preclinical studies were included when they provided mechanistic insights into inflammatory and immune pathways. Conference abstracts were included only when they reported data from ongoing or recently completed clinical trials not yet published in full.

## 2. Genetic Landscape of MPNs

Based on the WHO classification, classical MPNs consist of the Philadelphia chromosome-negative entities PMF, ET, and PV, which are caused predominantly through mutations in MPL, CALR, and JAK2 [5]. MPN-related mutations are divided into three categories: passenger mutations, clonal driver mutations, and disease driver mutations [18].

“Disease driver” mutations reproduce the human MPN phenotype when they are expressed in in vivo model systems [19]. A considerable number of MPN patients carry mutations in only one of the three disease driver genes, MPL, CALR, or JAK2, with no additional clonal driver mutations discovered by targeted next-generation sequencing (NGS) [18,20].

“Clonal driver” mutations do not induce the MPN phenotype when expressed in animal models, but they do so when combined with one of the “disease driver” mutations [18]. Notably, several “clonal driver” mutations affect blood counts, improve hematopoietic stem cell (HSC) fitness or proliferation rate, and cause pre-malignant alterations to hematopoiesis that are not strictly connected to MPN. The “clonal driver” genes which are most altered in MPNs consist of EZH2, DNMT3A, ASXL1, and TET2 [18]. Mutations in these genes are not particular to MPN and might be identified in all myeloid neoplasms. The MPN “disease driver” mutations, specifically JAK2-p. V617F, may also serve as “clonal drivers” in the case of clonal hematopoiesis without overt MPNs [21,22]. In conclusion, “passenger” mutations are sequence changes with no functional consequences. They might be used as markers to identify and track the subclonal organization in MPN [21]. In contrast, most patients with MPN carry distinct somatic mutations in one of three key driver genes: MPL (exon 10 of the thrombopoietin receptor gene), CALR (a frameshift mutation in exon 9 affecting calreticulin), or JAK2 (typically in exon 12 or 14) [22,23]. In recent years, numerous additional so-called atypical driver mutations have been found in the MPL and JAK2 genes of triple-negative MPN patients [5,21,22,23].

### 2.1. JAK2

JAK2 is the primary signaling component for erythropoietin, granulocyte-CSF, and thrombopoietin receptors [24] The JAK2V617F mutation, present in over 95% of PV and 50–60% of ET and PMF patients, constitutively activates the JAK–STAT pathway [24,25,26].Critically for this review, JAK2 activation directly promotes inflammation through two interconnected mechanisms: it increases NF-κB-dependent transcription of pro-inflammatory cytokines (TNF-α, IL-1β, IL-6) and enhances reactive oxygen species (ROS) production, creating a self-sustaining inflammatory cycle [8,27]. NF-κB signaling also polarizes macrophages toward pro-inflammatory M1 phenotypes and promotes monocyte activation [24]. This JAK2-NF-κB inflammatory axis is central to MPN pathogenesis, contributing to the cytokine storm immune dysregulation, and niche remodeling discussed in subsequent sections.

### 2.2. MPL

MPL encodes the thrombopoietin receptor and is central to MPN pathogenesis, as both MPL and CALR mutations converge on MPL-JAK2 signaling. JAK2V617F requires interaction with homodimeric type I cytokine receptors, including MPL, to drive disease development [28]. MPL regulates megakaryopoiesis, platelet formation, and hematopoietic stem cell maintenance through thrombopoietin signaling. MPL signaling depends on JAK2 association via Box 1 and Box 2 motifs in the intracellular domain. MPL is pre-associated with JAK2 in the endoplasmic reticulum, where JAK2 stabilizes MPL and facilitates its surface trafficking [29,30,31]. Upon ligand binding and receptor dimerization, JAK2 transphosphorylation activates downstream signaling cascades [29,31].

### 2.3. CALR

Subsequent studies found somatic mutations in the JAK–STAT pathway in JAK2V617F-negative MPNs, mostly found in the CALR gene [27,32]. CALR is an endoplasmic reticulum (ER) chaperone protein that regulates protein folding and calcium homeostasis [33]. CALR mutations generate a mutant protein with a unique C-terminal sequence that acts as a rogue chaperone, binding and stabilizing MPL to constitutively activate JAK2 signaling. Mutant CALR proteins possess a frame-shifted C-terminus that can bind to MPL, promote its dimerization and stabilization, before exporting it out of the ER [33]. Binding of mutant CALR to MPL extracellular domains causes MPL intracellular domains to be closely opposed to JAK2 C-terminal domains thereby activating JAK2. Genetic data indicate that CALR mutants’ capacity to generate autonomous hematopoietic progenitor development is dependent on cell surface location. Therefore, CALR mutants operate as rogue chaperones, bringing both functional and immature MPL molecules to the surface [33]. Importantly for this review, mutant CALR directly contributes to inflammation through two mechanisms: (1) it functions as a secreted extracellular “rogue cytokine” that activates inflammatory signaling in neighboring cells, and (2) JAK2 activation downstream of mutant CALR upregulates NF-κB-dependent inflammatory genes.

In summary, MPL, JAK2V617F, and CALR mutations use the MPL-JAK2 axis to cause MPN. Variations occur depending on the specific cellular compartment and the associated level of MPL-JAK2 pathway activation. This includes a stronger reliance of JAK2V617F on the PI-3-kinase pathway, various levels of MPL down-modulation, and differential utilization of STAT proteins [29,34,35]. Despite their role in continuous MPL-JAK2 signaling, CALR mutations critically influence the formation of a pro-inflammatory milieu by increasing cytokine production and activating downstream inflammatory pathways [34].

### 2.4. Other Mutations

Concurrent mutations reported in MPN clones primarily affect the SF3B1, TET2, SRSF2, EZH2, ASXL1, and DNMT3A genes. These mutations are classified as ‘clonal driver’ mutations, as they do not induce the MPN phenotype when expressed alone in animal models, but they significantly enhance hematopoietic stem cell fitness and promote clonal expansion when combined with disease driver mutations (JAK2, CALR, or MPL) [19]. These mutations are not unique to MPNs and are also found in other myeloid neoplasms and age-related clonal hematopoiesis. They affect up to 20% of PV, 20% of ET, and 40% of PMF cases. They often arise after acquiring a driver mutation, although they may also appear as initial events that favor clonal development, resulting in the acquisition of mutation(s) in JAK2, CALR, or MPL [36,37].

**Figure 1:**
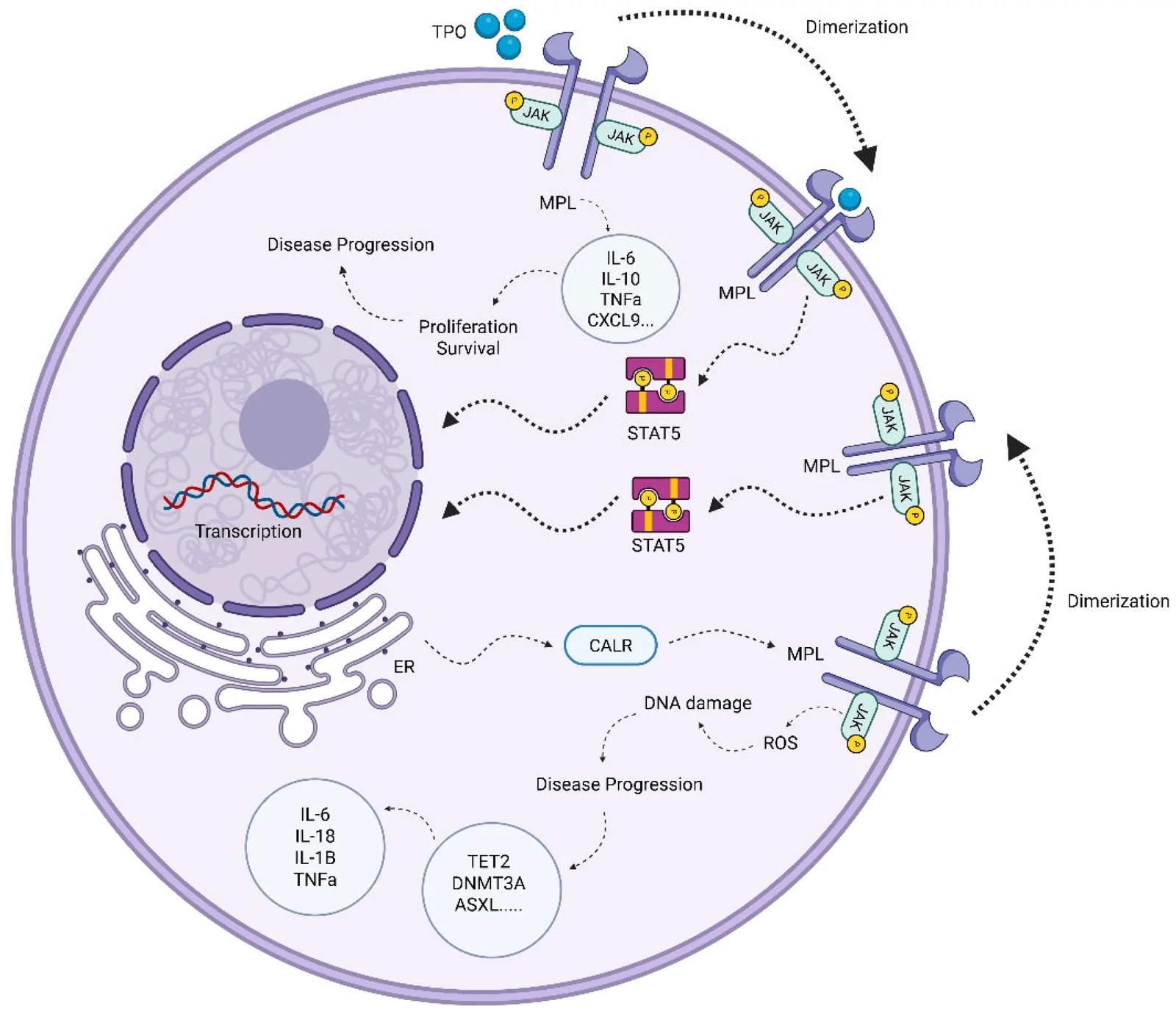
Role of CALR/MPL/JAK2 signaling axis and inflammatory pathways in MPN pathogenesis. TPO binding to MPL induces receptor dimerization and JAK2 activation, leading to STAT5 phosphorylation and nuclear translocation. Mutant CALR promotes MPL dimerization and stabilization, driving constitutive JAK2 activation. Aberrant CALR/MPL/JAK2 signaling enhances inflammatory cytokine production (IL-6, IL-1β, TNF-α, IL-10, IL-18, CXCL9), ROS accumulation, and abnormal hematopoietic cell survival. Epigenetic regulators (TET2, DNMT3A, ASXL1) further amplify inflammatory signaling, contributing to MPN progression. Created in BioRender. Keyhani, A. (2026) https://BioRender.com (accessed on 11 August 2026).

While these mutations do not directly induce the MPN phenotype, they are linked to clonal growth and progression of disease, particularly secondary myelofibrosis and leukemic transformation [38,39]. EZH2, TET2, ASXL1, and DNMT3A mutations affect epigenetic regulation and are more prevalent in PMF than PV and ET. Importantly for this review, TET2 and DNMT3A mutations activate inflammatory pathways, including NF-κB signaling. ASXL1 mutations in PMF have been linked to higher white blood cell counts and lower survival rates. SF3B1, TET2, SRSF2 and DNMT3A mutations may contribute to increased inflammation in subsets of MPN patients who carry these mutations. Of note, inflammation linked to concomitant mutations may precede the acquisition of mutations in the JAK2/MPL/CALR genes [36,37,40]. Taken together, driver and cooperating mutations work synergistically to promote clonal proliferation while reshaping the inflammatory and immunological landscape of MPNs. Chronic activation of JAK–STAT, NF-κB, and related signaling pathways causes abnormal cytokine production, which results in disease progression, fibrosis, thrombosis, and immunological dysfunction. As a result, understanding inflammatory cytokine networks is critical for understanding MPN etiology and developing new treatment targets (Figure 1) [36,40,41].

## 3. Inflammatory and Cytokine Networks in Myeloproliferative Neoplasms

Increasing evidence highlights the central pathogenic role of inflammation in both the initiation and progression of MPNs [12,42,43,44,45]. Chronic inflammation also contributes to the development of major clinical manifestations, including pulmonary hypertension, splenomegaly, thrombosis, constitutional symptoms, bone marrow fibrosis, and anemia, thereby increasing disease-related morbidity and mortality [12,42,43,45].

Chronic non-neoplastic inflammation resulting from autoimmune disorders or persistent infections leads to sustained production of pro-inflammatory cytokines, including IL-8, TNF-α, and IL-6, accompanied by the accumulation of ROS [43]. These processes promote malignant transformation and clonal expansion by inducing genetic instability, oxidative stress, resistance to apoptosis, and alterations in cellular migration [43,44].

Furthermore, some investigators propose that MPNs exemplify an inflammatory model of tumorigenesis, as evidenced by aberrant cytokine synthesis and a link to various inflammatory/autoimmune disorders and secondary malignancies [4,43]. In this paradigm, chronic inflammation is proposed to induce the initial oncogenic event in hematopoietic stem cells (HSCs), promoting mutagenesis and genomic instability that ultimately contribute to MPN development [4,46]. Conversely, mutations such as JAK2V617F may arise stochastically in aging bone marrow. Oncogenic lesions trigger inflammatory pathways in HSCs and progenitor cells, leading to the generation of ROS and pro-inflammatory cytokines [4,46,47]. During the early stages, the disease may remain clinically silent and manifest only as clonal hematopoiesis accompanied by a subclinical inflammatory state. The buildup of ROS in mutant cells breaks DNA and promotes clonal proliferation, leading to the development of overt MPN [4,46,47]. The JAK2V617F mutation increases the expression of various cytokines, chemokines, and growth factors, such as IL-1β, IL-33, IL-8, IL-15, IL-11, IL-17, IL-10, IL-12, IL-6, CXCL1, VEGF, CXCL4, TNF-α, GM-CSF, TGF-β, PDGF, and angiopoietin-1, as seen in murine models and patient samples [4,46,48]. Stimulation of the HIF-α and NF-κB signaling pathways increases the synthesis of soluble mediators, leading to a self-sustaining inflammatory cycle driven by stimulated neutrophils, platelets, and monocytes [4,46,47,49].

Each inflammatory cytokine has distinct biological effects. For example, IL-8 has been linked to constitutional symptoms and leukemic change in patients with PMF [4,48]. Furthermore, every type of MPN has a distinct cytokine profile, including preferential activation of certain inflammatory mediators. These differences may influence disease phenotype, symptom burden, prognosis, and the immune cell composition, and hematopoietic lineage involvement [4,47,48].

Table 1 and Table 2 summarize the biological functions of individual cytokines as well as the cytokine profiles associated with each MPN subtype [46].

The distinct cytokine profiles observed across MPN subtypes have potential utility in clinical practice. Elevated IL-8, IL-2R, and CXCL10 levels have been independently associated with reduced survival in PMF [73], while increased IL-1RA and HGF correlate with severe splenomegaly [53,57]. Furthermore, elevated TNF-α and IL-6 levels may serve as biomarkers of disease activity and therapeutic response [62,67]. From a therapeutic standpoint, these cytokine signatures provide a rationale for combining JAK inhibition with targeted anti-inflammatory agents. For instance, IL-1β-driven clonal expansion of JAK2-mutant HSCs suggests that anti-IL-1β therapies could complement JAK inhibition in early disease stages [61]. Similarly, elevated IL-13 and TGF-β in fibrotic states support investigation of anti-fibrotic agents targeting these pathways [55,60]. Cytokine profiling may therefore aid in risk stratification, monitoring disease progression, and guiding personalized combination therapy approaches (Table 2).

## 4. Remodeling of the Immune Cell Compartment in Myeloproliferative Neoplasms

Cancer formation is based on the capacity of neoplastic cells to evade immune surveillance within the TME [12]. In MPNs, mutant hematopoietic stem cells help to maintain an inflammatory TME by promoting clonal myeloproliferation and inhibiting cytotoxic T cells and other antitumor defenses. Chronic inflammation and immunological dysregulation are associated with abnormalities in lymphocyte subsets, such as B cells, T cells, and NK cells [74]. Additional quantitative and functional deficiencies in PMF affect Th1, Th17, NK, and innate lymphoid cells, as well as a diminished ability of monocytes to develop into dendritic cells. Furthermore, loss of HLA-II expression and dysfunctional dendritic cells impair T cell priming and can lead to the development of an MPN-like disease [4,68].

### 4.1. Adaptive Immune Exhaustion and Immune Surveillance Failure

MPNs carrying the JAK2V617F mutation are characterized by a progressive decline in absolute lymphocyte counts that is greater than expected with normal ageing and seems to be independent of cytoreductive therapy. Experimental studies suggest that such lymphopenia is at least in part due to loss of NOTCH signaling, a critical signaling pathway for lymphocyte development, resulting in an inability to develop lymphoid lineages. As mutant hematopoietic stem cells gradually outcompete normal stem cells, lymphopoiesis increasingly relies on a dwindling pool of healthy progenitors, which drives immune dysfunction. The mutation also drives a myeloid-biased hematopoiesis at the expense of lymphoid commitment, resulting in reduced lymphocyte production. This effect may be even more pronounced in a bi-allelic disease. Therefore, lymphopenia increases the neutrophil-to-lymphocyte ratio, a parameter linked to immune dysregulation and poor outcomes in MPNs [4,75]. The discovery of clonal T and B cells (which carry JAK2 or CALR mutations) indicates that certain lymphocyte subpopulations have intrinsically impaired immunological capabilities. Furthermore, paracrine actions of extracellular mutant CALR peptide in the TME may inhibit cancer cell phagocytosis and contribute to antitumor evasion [12,76].

B cells that produce IL-10 regulate T cell responses and decrease autoimmunity. These cells have IL-10-independent impacts, including the expression of PD-L1 (PD-L1-expressing B cells), which modulates follicular helper T cells (CD4^+^CXCR5^+^PD-1^+^) and decreases inflammation. Additionally, these cells are insensitive to anti-CD20-induced suppression and have an important role in regulating humoral immunity [77]. In contrast to the usual IL-10-producing regulatory B cells, these PD-1+ B cells mainly inhibit the proliferation and survival of CD4+ and CD8+ T cells through PD-L1-dependent pathways; furthermore, PD-1+ B cells are uncommon in peripheral blood but greatly increased in differentiated neoplasms such as thyroid tumors, expressing high levels of PD-L1. These results emphasize their function in regulating T-cell responses within the TME [4,78].

In MF, T cells are more activated and exhausted and express more PD-1 in their CD4+ and CD8+ subtypes than in healthy people. T cells transition from an inactive state to activation by acquiring an effector phenotype. JAK inhibitor ruxolitinib normalizes these abnormal T cell patterns to resting phenotypes. CD4+ and CD8+ subsets have baseline levels that correlate with platelet and monocyte counts, whereas their PD-1+ fractions associate with leukocyte numbers and the size of the spleen. Reduced concentrations of exhausted PD-1+ CD4+ and CD8+ T cells are connected to reduced spleen volume and better survival rates, indicating that lower T cell exhaustion anticipates more favorable treatment results [79].

The frequency of PD-L1+ and PD-1+ B cells in MF and PV patients is higher than in healthy subjects, suggesting that these regulatory B-cell types could be contributing to immune suppression in MPNs. PD-L1+ and PD-1+ B cells decrease after therapy, implying that immunological function is restored, which may boost antitumor immunity and mitigate disease-related immune system disorders [80].The expression of CD27 plays a role in the differentiation between regulatory and effector T cells within the CD4+CD25+ subset in inflamed settings. CD4+CD25+CD27+ cells are a regulatory subtype of T cells (Tregs) that are highly FoxP3+, do not synthesize pro-inflammatory cytokines and strongly inhibit T cell proliferation. In contrast, CD4+CD25+CD27− cells are effector T cells with decreased FoxP3 expression, increased cytokine production, and a lack of suppressive activity [4].

Th17 cells perform complex and sometimes opposing activities in tumor biology. They can promote tumor growth by stimulating angiogenesis, maintaining chronic inflammation, and allowing immune evasion via IL-17-mediated neutrophil recruitment and myeloid-derived suppressor cells. Th17 cells can develop into IFN-γ-producing Th1-like cells, improving cytotoxic T cell activity and tumor rejection, depending on the molecular context [4,81].

Short-term therapy reduces CD3+ T cells, Th1, Th17 cells, and Tregs, as well as the production of pro-inflammatory cytokines such as IL-1β, IL-5, IL-6, and TNF-α. These data indicate a broad immunomodulatory influence on CD4+ T cell function [82]. According to reports, JAK inhibitors initially reduce CD4+ T cell activity and cytokine production before causing an immunological profile shift that may favor a Th17-dominant reaction. This combined effect may contribute to the higher incidence of atypical infections found in some patients receiving treatment [4,83].

JAK2V617F, CALR, and triple-negative MF alterations impact immune cell subsets. All have reduced dendritic cells and inadequate monocyte to DC development. Studies showed that patients with JAK2 mutations had higher levels of ILC1 and cytokine-producing Tregs but lower levels of Th17, myeloid DC, and effector Tregs. CALR mutant patients have increased ILC3, decreased Th1 cells, and functionally impaired Tregs with enhanced proliferation. In triple-negative patients, CD3+ T cells, effector Tregs, and Th1 cells are decreased, but ILC1 is increased. These observations suggest mutation-specific immune dysregulation, but bigger cohort studies are required for validation [4,47].

Studies of immunological changes in MPN included analysis of lymphocyte subsets by flow cytometry, with special attention to the link between immune components and DARS expression. DARS, which encodes aspartyl-tRNA synthetase, is negatively associated with circulating CD4+ T cell counts and the CD4+/CD8+ T cell ratio. High DARS expression was associated with decreased CD4+ T cells, which are required for B cell, macrophage, and cytotoxic CD8+ T cell activation, but CD8+ T cell antitumor efficacy is limited in the absence of appropriate CD4+ T cell support. This decline is indicative of an altered immune function which may compromise antitumor responses and contribute to disease progression. These findings imply that abnormal DARS expression may play a role in the depletion of CD4+ T cells in the MPN microenvironment and require additional mechanistic investigation [74].

Tregs are not consistently expanded in MPNs but rather have an inconsistent and dysregulated pattern. In untreated patients, their frequency and phenotype are usually comparable to those of healthy individuals, but functional studies indicate that Tregs might be impaired with reduced suppressive capacity despite altered cytokine production [12]. JAK inhibition with ruxolitinib has been reported to result in a decrease in circulating Tregs, which may partially impair immune surveillance and could contribute to the increased incidence of infections [4].

### 4.2. Defective Antigen Presentation and Neoantigen-Specific Immunity

Analysis of immune-related gene expression in MPNs showed decreased transcription of HLA class I and class II molecules and other HLA-associated genes such as CD40L and FAS [12]. These alterations are associated with abnormal antigen presentation, impaired APC co-stimulatory signaling, and decreased T cell cytotoxic function. Experimental investigation in mouse models showed that the combined deficiency of HLA-II and CD4+ T cells greatly diminished the development of MPN. This suggests that unprimed pro-inflammatory CD4+ T cells could be required for the establishment of an MPN-permissive environment [12,74,84]. Neoantigens, which are non-self-epitopes generated from somatic mutations that are presented by HLA molecules and recognized by T cell receptors, are considered highly immunogenic targets for cancer immunotherapy. In hematologic malignancies, neoantigen-specific T cell responses have been reported in leukemias and multiple myeloma and are associated with clinical outcomes, forming the basis for ex vivo expanded T cell–based immunotherapies [12]. In MPNs, ex vivo studies using ELISpot, flow cytometry, and cytotoxicity assays have identified “MPN-specific” T cells targeting JAK2V617F and CALR exon 9 mutations, including CD4+ Th1 and CD8+ T cells capable of recognizing and eliminating mutant cells. These responses are accompanied by exhaustion markers such as PD-1 and CTLA-4, although partial functional reversibility has been reported, and similar responses can also be detected in healthy individuals, suggesting the presence of immune surveillance that is weakened in patients with MPNs. In addition, T cell responses against PD-L1 and ARG1 have been observed in a substantial proportion of cases, with higher frequencies in non-advanced disease, while potential neoantigens also arise from abnormal splicing events (e.g., CALR/MPL variants linked to SF3B1 mutations). Overall, JAK2 and CALR mutations generate shared highly immunogenic peptides across patients, together with additional tumor-associated immune targets, supporting their relevance for adoptive T cell therapy and vaccination strategies [12,85,86]. The identification of neoantigen-specific T cell responses targeting CALR mutations has directly informed the development of CALR-directed immunotherapies discussed in Section 6. Specifically, these findings provide the biological rationale for CALR peptide vaccines (CALRLong36, mutant-CALR peptide vaccine, VAC85135), bispecific T-cell-redirecting antibodies (JNJ-88549968, INCA035784), and CALR-targeted monoclonal antibodies (INCA033989), all of which aim to harness or enhance neoantigen-specific T cell immunity against mutant clones. Moreover, the presence of exhaustion markers on these neoantigen-specific T cells supports the rationale for combining CALR-directed immunotherapies with immune checkpoint inhibitors, as exemplified by the VAC85135 plus ipilimumab combination trial. This connection between immune surveillance failure and therapeutic targeting underscores the clinical relevance of understanding neoantigen-specific immune responses in MPNs.

### 4.3. Natural Killer Cell Dysfunction and Immune Escape in MPN

In addition to defects in adaptive immunity, the innate immune compartment was discovered to be attenuated in MPNs [12]. NK cells, which are innate lymphocytes, play critical roles in tumor defenses by secreting cytokines, including IFN-γ and TNF-α, which affect the development and activity of immune cells. Therefore, understanding NK-cell phenotype and function and how they contribute to pathogenesis, immune escape from malignancy, and MPN surveillance may lead to new treatments [87,88]. These cells are essential for the regulation of metastatic spread, as low NK cell counts are associated with advanced disease and a poor prognosis, whereas robust NK cytotoxicity is associated with improved outcomes. NK cells are divided into two primary subsets: CD56 dim CD16+ (cytotoxic, comprising approximately 90% of peripheral blood NK cells) and CD56 bright CD16− (modulatory, cytokine-producing). The NK cell counts of patients who were not treated with MPNs were remarkably low [4,89]. Studies have demonstrated that, although the absence of an absolute reduction in NK cells, individuals with JAK2VF mutations have poor NK cell maturation, with an increase in immature NK cells and a decrease in mature cytotoxic NK cells. MPN patients have a higher number of NK cells based on the CD56/CD16 pattern, but their maturation profile is imbalanced, with more non-cytotoxic CD11b− cells and fewer mature CD11b+ cells. CD11b and CD27, markers of NK cell maturation in human NK cells, were used to identify human NK in MPN patients [89]. Both CD11b+ and CD11b− populations were reduced in PV and ET compared to healthy individuals, whereas PMF patients not only demonstrated a less cytotoxic phenotype but also transitioned to an immature NK phenotype, with a significant increase in CD11b− and CD11b−CD27+ NK cells. Thus, PMF samples and primary NK cells with JAK2 mutations demonstrated a more secretory NK cell behavior, which may contribute to the disease’s significant inflammatory character [89]. Practically, despite enhanced degranulation (increased levels of CD107a), MPN patients’ NK cells showed reduced cytotoxicity potential. The expression of CD107a is associated with the production of cytokines and the lysis of target cells by NK cells. Thus, MPN patients exhibit a phenotype that is immature and secretory, which may promote degranulation but fails to eradicate the target cell. Furthermore, the data showed that JAK2V617F NK cells are less responsive to activation (due to reduced expression of CD69 and CD107a functional biomarkers) and proliferation following stimulation, implying that, in addition to maturation arrest, NK cells are ineffective in MPN.

Adequate NK cell numbers and functional maturation are required to maintain normal HSC activity and regulation. The arrest of NK cell development in MPN may contribute to disease progression. To confirm this, normal HSCs were cultivated with two types of NK cells: normal NK cells (JAK2WT) and NK cells carrying a functionally deficient JAK2 mutation. The findings indicated that normal NK cells were better capable of suppressing HSC colonization. In contrast, NK cells with a JAK2 mutation exhibited a less inhibitory impact and resulted in a lower decrease in colony counts. This demonstrates that NK cell phenotypic and functional abnormalities in MPN can directly affect HSC regulation and disease progression [89,90].

### 4.4. Myeloid Immune Suppression: MDSCs, Monocytes, and Macrophages

In addition to Tregs, various cell-dependent immunosuppressive mechanisms have been proposed for MPN immune evasion from antigen-specific T-cell responses. Specifically, myeloid-derived suppressor cells (MDSCs) may constitute an essential connection between inflammation and the suppression of antitumor T cell immunity; they have previously demonstrated significant activity in several hematologic neoplasms. In MPNs, CD11b+CD14-CD33+ cells (MDSCs) were considerably more common in patients than in controls, and they were associated with increased production of arginase-1 (ARG1) mRNA and immunosuppressive activity towards autologous T cells [12,91,92]. MPN-related clonal thrombocytosis may also have a “platelet-cancer loop” where pathologic platelets inhibit specific T cells through TGF-β secretion [12].

Mature hematopoietic cells in the peripheral circulation are the major source of increased systemic cytokines in MPN. In MF, classical CD14+CD16- monocytes generate the greatest quantities of cytokines such as TNF-α, IL-6, IL-8, and IL-10. TNF-α levels remain increased in all MPN subtypes, leading to clonal proliferation of JAK2V617F mutant cells. The investigation found that the primary monocytes in MPN cases release more TNF-α following stimulation than those from healthy individuals, indicating a decreased responsiveness to the anti-inflammatory cytokine IL-10. IL-10R signaling via the inhibitor of cytokine signalling-3 (SOCS3) pathway has been shown to be diminished in MPN cases. Both non-mutated and JAK2V617F-mutated monocytes produced persistent TNF-α, indicating that monocytes play a non-cell autonomous role in MPN inflammation. Also, CD56+CD14+ inflammatory monocytes are a primary source of GRO-α in ET patients, which promotes MPN disease development [59,68,93]. Notably, fibrocytes have been detected in the bone marrow of PMF patients. These cells contribute to the deposition of extracellular matrix (ECM) and are involved in the fibrotic process. These results indicate that monocyte-derived fibrocytes play an important role in disease development [4,94].

Macrophages in the bone marrow have a role in hematopoiesis regulation including the clearance of myeloid cells and discarded nuclei in erythropoiesis [95], and their higher presence in PMF compared to PV and ET is most likely due to enhanced cell turnover, particularly in the severe granulopoiesis of PMF. However, it is unclear if this rise is a cause or a result of the disease, given all MPNs share common mutations (JAK2, CALR, MPL). It is also likely that macrophages are part of the malignant clone, with M2 macrophages playing a critical role in the genesis and progression of fibrosis by secreting profibrotic substances [96,97].

Tumor-Associated Macrophages (TAMs) are an important immune-cell population in the TME. They have been found to be closely related to tumor progression, and substantial TAM infiltration in tumors is a predictor of poor prognosis in cancer patients [98]. MPN patients exhibiting elevated DARS expression had a considerably higher disease burden, and CD68 expression associated favorably with DARS expression. This shows that CD68-positive TAMs may play a role in MPN progression. This discovery is consistent with previous studies on TAMs in hematological neoplasms, for instance, acute leukemias and lymphoma. Notably, TAMs can impair the activity of CD4+ and CD8+ T cells by producing immunosuppressive substances. In brief, elevated CD68 expression in MPNs may indicate an immunologically suppressive TME [74].

MPNs have an increased percentage of CD163+ monocytes/macrophages as well as the CD14+CD16+ subpopulation in the bone marrow, which is associated with various disease subtypes. In PV patients the percentage of CD163+ monocytes/macrophages correlate positively with HGB levels, as does the CD14+CD16+ subpopulation. In ET patients the percentage of CD163+ monocytes/macrophages correlate positively with PLT, whereas the CD14+CD16+ subpopulation does not. In PMF patients the percentage of CD163+ monocytes/macrophages correlate negatively with HGB, as does the CD14+CD16+ subpopulation. Overall bone marrow monocyte/macrophage changes are associated with various clinical phenotypes of MPN [99].

Investigations showed that frequency profiles of Macrophage and mast cell in BM biopsies normalize after JAK1/2 inhibition in a considerable proportion of PMF patients, accompanied by lower grades of fibrosis [96].

### 4.5. Thrombo-Inflammation: Neutrophils, NETosis, and Platelets

Neutrophils perform an important pro-inflammatory and pathogenic role in MPN. JAK2V617F-mutated neutrophils increase inflammation by generating cytokines (including IL-1β and IL-8), exhibiting resistance to apoptosis, and promoting disease development. The transcription factor NF-E2, which is increased in MPN, is activated in neutrophils, increasing IL-8 secretion and contributing to neutrophilia and hematopoietic stem cell mobilization; neutrophils are also an important source of IL-8 in myelofibrosis. Biomarkers, including NGAL (lipocalin-2), YKL-40, those associated with neutrophil count, and JAK2V617F mutant load, are increased, and NGAL enhances malignant stem cell survival while worsening fibrosis [4,100]. In MPN, neutrophils with the JAK2V617F mutation (as opposed to CALR) produce more pro-inflammatory cytokines and evade immune clearance by overexpressing the CD24 (phagocytosis-inhibitory) signal in the GM-CSF-JAK2-STAT5 pathway. CD24+ neutrophils infiltrate megakaryocytes via emperipolesis, leading to increased TGF-β production, platelet formation, and the development of myelofibrosis [4].

Neutrophils play a crucial role in JAK2V617F MPN-associated thrombosis, and platelets are required to enhance the activation of neutrophils and NETosis [101]. Neutrophil extracellular traps (NETs) are network-like structures made of antimicrobial proteins, enzymes, nucleic acids, and histones that neutrophils release to catch and eradicate pathogens. Although NETs serve a vital role in host defense and innate immunity, they may also cause sterile inflammation. NETosis is the process by which decondensed DNA, nuclear proteins, and enzymes such as myeloperoxidase are released into the extracellular environment [102]. In addition to their antibacterial role, NETs have been demonstrated experimentally to cause platelet aggregation and clustering, particularly following activation with lipopolysaccharide. These findings emphasize the importance of neutrophil-dependent processes in thrombus development [4,101]. Platelets have a crucial role in the development of the thrombo-inflammatory state in MPN, in addition to their hemostatic function [103]. Thrombosis is a leading cause of death and morbidity in these patients, and multiple processes are involved, notably red blood cell membrane alterations, formation of platelet-leukocyte aggregates, platelet and leukocyte activation, and endothelial defects. A systemic inflammatory response also contributes to an increased risk of thrombosis, with higher hsCRP levels related to thrombotic events in PV and ET [68,104].

**Figure 2:**
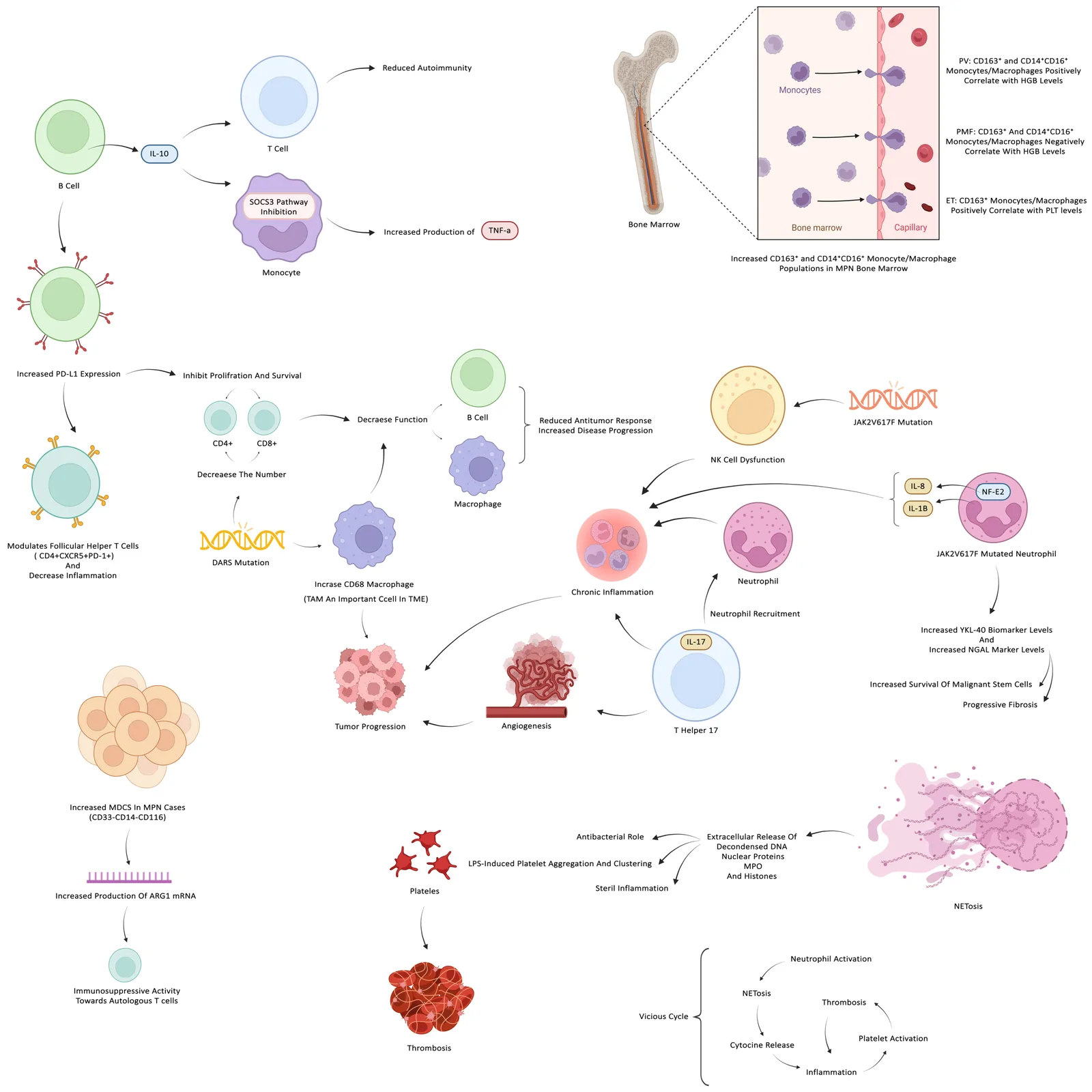
Immune dysregulation and cellular interactions in MPNs. B cells exert immunomodulatory effects through IL-10 production and PD-L1 upregulation, modulating T-cell responses, monocyte function, and inflammation. DARS mutation is associated with reduced CD4^+^ and CD8^+^ T-cell populations and increased CD68^+^ macrophage abundance, potentially impairing immune function and promoting disease progression. The JAK2V617F mutation induces NK-cell dysfunction and chronic inflammation. Th17 cells promote neutrophil recruitment, inflammation, and angiogenesis. JAK2-mutated neutrophils contribute to inflammation through IL-1β and NF-E2/IL-8 signaling, while increased YKL-40 and NGAL expression is associated with fibrosis and malignant stem-cell survival. Neutrophils also contribute to NETosis and, together with platelets, promote a pro-inflammatory and prothrombotic environment. MDSCs suppress T-cell activity through increased ARG1 expression. Distinct monocyte and macrophage subsets are expanded in the bone marrow across MPN subtypes and may contribute to differences in disease phenotype and clinical manifestations. Created in BioRender. Keyhani, A. (2026) https://BioRender.com (accessed on 11 August 2026).

Platelet interaction with monocytes and neutrophils stimulates these cells, exacerbating the inflammatory and thrombotic mechanisms. Furthermore, the production of NETs in the presence of MPN platelets implies the emergence of a prothrombotic milieu. Platelets and monocytes both boost the production of inflammatory cytokines, whereas cytokines and ROS activate the endothelium, resulting in a prothrombotic phenotype marked by increased expression of E-selectin and von Willebrand factor (vWF). Ultimately, the attraction of platelets and inflammatory cells to the activated endothelium aggravates the condition [68,105]. Activated platelets also function as immune cells, causing inflammation by secreting cytokines, which include PF4 (CXCL4) and CCL5 through alpha granules. Eventually, elevated cytokines cause more platelet activation, resulting in a thrombo-inflammatory vicious cycle in MPN [68,106].The profound immune dysregulation observed in MPNs has direct implications for immunotherapy development and patient management. The presence of exhausted PD-1+ T cells and elevated PD-L1+ B cells provides a rationale for testing immune checkpoint inhibitors, although clinical data in MPNs remain limited [79,80]. The identification of neoantigen-specific T cells targeting JAK2V617F and CALR mutations supports the development of peptide vaccines and adoptive T-cell therapies [85,86]. However, the concurrent accumulation of MDSCs and impaired NK cell maturation may limit the efficacy of such approaches, suggesting that combination strategies incorporating MDSC depletion or NK cell agonists may be necessary [12,92]. Furthermore, mutation-specific immune profiles (e.g., lower Th17 and myeloid DC in JAK2-mutated patients vs. decreased Th1 in CALR-mutant patients) could guide patient selection for immunotherapeutic trials [47]. Monitoring lymphocyte subsets and exhaustion markers may also help predict treatment responses to JAK inhibitors, as reduced exhausted T-cell fractions correlate with better spleen responses and survival [79]. These immunological parameters should be considered in future trial designs and may eventually inform personalized immunotherapy strategies in MPNs (Figure 2).

## 5. Bone Marrow Niche Remodeling in MPNs

The bone marrow microenvironment is a dynamic structure that plays an important role in the survival and development of hematopoietic stem cells. In myeloproliferative neoplasms, this niche is impacted by persistent inflammation and immunological responses, as well as the altered cells’ genetic backgrounds. Mutated stem cells and niche components can interact, leading to disease development and medication resistance [107].

MPN HSC behavior can change under the influence of host and external factors. As the cancerous clone grows, MPN HSC expansion surpasses normal HSC proliferation [108]. JAK2V617F-LT-HSC can initiate and promote the condition, giving them a clonal advantage in dominating the niche over WT cells. Furthermore, mutant LT-HSC may induce fibrotic alterations in the bone marrow niche in WT implanted animal models [107,109]. High expression levels of JAK2 have a deleterious influence on the repopulation ability of LT-HSC and increase disease expansion [110]. Acquisition of additional mutations, such as deletions of TET2 in JAK2V617F-LT-HSC, provides a clonal advantage favoring disease development [107,111].

In myeloproliferative neoplasms (MPNs), disturbance of the osteoblast-osteoclast axis, particularly the endosteal osteoblastic niche, is crucial for disease persistence and progression. MPN induces fibrosis by altering the functional growth of osteoblasts. Disease-driven endosteal niche remodeling happens over time, resulting in a self-reinforcing ‘leukemia-niche’ with compromised normal hematopoietic function. Multiple mechanisms have been implicated in aberrantly regulated osteoblastic growth, including monocytes produced from JAK2V617F (heterozygote)-MPN cells, which were more capable of becoming osteoclasts than wild-type monocytes. An enhanced osteoclast environment also promotes MPN-associated mutant cell population development and survival [107,112,113,114].

Mutant clonal cells in MPNs disrupt the balance of the bone marrow’s vascular niche, creating a favorable environment for survival. JAK2-mutated endothelial cells promote mutant HSC proliferation and survival while inhibiting normal HSC function via CXCL12 and SCF pathway changes. Furthermore, endothelial cells and mutant HSCs produce VEGF, which promotes angiogenesis, survival, and proliferation of clonal cells, resulting in a self-sustaining vascular niche [107,115].

BM mesenchymal stem cells (BMSCs) are crucial for the development and sustenance of the MPN phenotype. BMSCs drive the abnormal proliferation of osteoblasts as inflammatory ‘myelofibrotic’ cells. This transformation is precipitated by dysregulation of inflammatory signaling pathways characterized by high levels of TNF-β, TGF-β1, IL-6, IL-1β, and Notch. These changes result from direct interaction between clonal MPN-HSC and BMSCs [107,116].

These niche alterations are interconnected through inflammatory cytokines (TGF-β, IL-6, IL-1β, TNF-α) that mediate crosstalk between megakaryocytes, monocytes/macrophages, endothelial cells, mesenchymal stromal cells, and sympathetic nerves, collectively supporting mutant HSC survival, fibrosis, and therapy resistance [107,117].

In addition to the mechanisms discussed above, there are many factors and pathways that influence the bone marrow niche, each of which alters the course of the disease and causes variances in response to therapy. However, the emerging picture is that inflammation serves as the common thread linking all niche compartments from osteoblasts to endothelial cells to mesenchymal stromal cells into a unified pathological network. This inflammatory-niche axis represents a potential therapeutic vulnerability, and agents that target inflammatory cytokines (e.g., anti-TNF-α and anti-IL-6), modulate niche components, or disrupt the crosstalk between mutant cells and their microenvironment may offer additive or synergistic benefits when combined with JAK inhibition. Further characterization of these pathways may facilitate the development of more effective therapeutic strategies [107,117,118,119].

## 6. Therapeutic Implications of Targeting MPNs

### 6.1. JAK1/JAK2 Inhibitors as Approved MPNs Treatments

The pro-inflammatory state has become a primary focus of research, as evidenced by the fact that JAK1/JAK2 inhibitors are currently FDA-approved treatments for MPNs. The identification of the JAK2V617F mutation led to the creation of JAK inhibitors (JAKi), notably the first-in-class JAK1/JAK2 inhibitor ruxolitinib and later approvals of fedratinib, pacritinib, and momelotinib [120].

The FDA first approved the oral JAK1/JAK2 inhibitor ruxolitinib in 2011 to treat individuals with moderate or severe MF, including primary MF, post-polycythemia vera MF, and post-essential thrombocythemia MF. It has been established that ruxolitinib improves overall survival (OS) as well as splenomegaly and the symptom burden of MF. As a result, ruxolitinib remains a standard-of-care therapy for patients with more severe MF more than ten years after it was first approved [121]. Ruxolitinib’s drawbacks, especially in treating emerging cytopenias, led to the development of later-generation JAK inhibitors. Fedratinib is an effective oral JAK2 inhibitor that targets both mutant and wild-type JAK2 and FLT3. It was granted approval by the US FDA in August 2019 to treat adult patients with moderate or severe primary or secondary MF [122]. Fedratinib may address an important gap for patients who discontinued their use of ruxolitinib because of resistance or intolerance, since many people discontinue it within three to five years [122]. Additionally, in the case of severe thrombocytopenia, both fedratinib and ruxolitinib require dose adjustment or hold because they are linked to myelosuppression. Therefore, for patients having MF and a platelet count less than 50 × 109/L, the only non-SCT therapeutic treatment option has generally been to enroll in a clinical trial. This changed in February of 2022, after pacritinib was granted approval to treat individuals with MF and severe thrombocytopenia [123]. Pacritinib, a dual inhibitor of JAK2 and FLT3 that also inhibits IRAK1, has been shown to improve MF-related splenomegaly and symptom burden while causing minimal myelosuppression and manageable gastrointestinal toxicity [124].

While pacritinib improved the treatment paradigm by offering effective splenomegaly and symptom control with low myelosuppression, the ongoing unmet need for treatments that target MF-associated anemia ultimately led to the development and subsequent approval of momelotinib [122,125]. Momelotinib, an oral, small-molecule inhibitor of JAK1/2, and activin A receptor type 1 (ACVR1), was granted approval in the US in September of 2023 and the EU and other countries globally in 2024 for adults with moderate or severe MF with anemia [125]. The findings of the phase III MOMENTUM trial in JAK inhibitor-experienced patients and the phase III SIMPLIFY-1 trial in JAK inhibitor-naïve patients supported momelotinib’s regulatory approval. In these trials, momelotinib was linked to improvements in anemia-related measures, such as higher transfusion independence rates, and significant decreases in symptom burden and splenomegaly. While these agents have transformed symptom management and provided meaningful clinical benefit, the limitations of JAK inhibitor monotherapy including lack of disease-modifying activity, development of resistance, persistence of mutant hematopoietic stem cells, and continued disease progression in most patients have motivated the development of novel therapeutic strategies and combination approaches.

Given the central role of chronic inflammation in MPN pathogenesis, anti-inflammatory agents represent an important adjunctive therapeutic strategy. Low-dose aspirin is widely used in MPN patients for primary and secondary prevention of thrombotic events, particularly in PV and ET patients at high risk for thrombosis [126]. Aspirin irreversibly inhibits COX-1, reducing thromboxane A2 production and platelet aggregation; however, it does not address the underlying inflammatory cytokine storm and has limited effect on disease progression [126]. Beyond COX inhibition, the arachidonic acid pathway offers additional therapeutic targets. Lipoxygenases, particularly 5-LOX, are key enzymes in leukotriene biosynthesis, and elevated leukotriene levels have been detected in MPN patients [127]. Inhibitors of 5-LOX, such as zileuton, have been investigated in preclinical models of inflammatory hematologic conditions, though clinical data in MPNs remain limited (166). Other anti-inflammatory agents with potential relevance include colchicine, which disrupts microtubule polymerization and inhibits NLRP3 inflammasome activation, canakinumab (anti-IL-1β monoclonal antibody) which targets IL-1β-driven clonal expansion of JAK2-mutant HSCs [61], and NLRP3 inflammasome inhibitors such as MCC950 that reduce IL-1β and IL-18 production in preclinical models [128]. While these anti-inflammatory agents have shown biological plausibility, large-scale clinical trials specifically in MPN patients are lacking. Future studies should evaluate whether these agents, alone or in combination with JAK inhibitors, can provide additive anti-inflammatory and anti-thrombotic benefits, delay disease progression, or improve survival.

### 6.2. Clinical-Stage Investigational Therapies

The existing FDA-approved JAK inhibitors have substantial anti-inflammatory qualities, but they have not demonstrated a significant effect on the progression of the disease or overall survival [120]. As a result, research is now mainly centered on developing new treatments, either as monotherapy or in combination with other agents, especially JAK1/JAK2 inhibitors. Table 3 summarizes the agents that are currently being investigated.

Rovadicitinib (TQ05105) is a novel, oral small-molecule JAK/ROCK inhibitor that was approved in China in February 2026 as a first-line treatment for adults diagnosed with moderate- or high-risk PMF, PPV-MF, or PET-MF [145]. A multi-center, open-label, phase Ib trial was conducted to assess the safety and effectiveness of rovadicitinib in MF patients classified as intolerant or resistant to previous Ruxolitinib treatment (NCT06388759). Thrombocytopenia was the most frequent grade ≥ 3 treatment-emergent adverse event (TEAE) during a median treatment duration of 12.5 months (33.3%) [129]. Only one individual (11.1%) discontinued therapy, and no bleeding incidents were observed. These findings demonstrate that rovadicitinib can offer therapeutic benefit in individuals with MF who are resistant or intolerant of ruxolitinib or other JAK inhibitors, with spleen and symptom response rates of 25.0% and 37.5%, respectively [129]. However, these data are derived from a phase Ib trial with a limited sample size (*n* = 9), and larger confirmatory studies are warranted to validate these preliminary findings and to establish the long-term safety and efficacy profile of rovadicitinib in broader patient populations.

Epigenetic regulation is a promising field of therapeutic research, with several epigenetic modulators currently in preclinical and clinical trials. A promising class of medications known as inhibitors of bromodomain (BD) and extra-terminal domain (BET) proteins has shown promise in modifying important processes related to apoptosis, fibrosis, and inflammation. Pelabresib (CPI-0610) is a small molecule BET inhibitor that is being investigated in clinical studies for individuals diagnosed with myelofibrosis (MF). This agent has demonstrated promising clinical efficacy in its Phase III trial and is well tolerated as a monotherapy and in combination with the JAKi ruxolitinib, as discussed below [134]. ABBV-744 is a novel, selective BETi which targets the BDII rather than the BDI in the BET family of proteins at low doses. ABBV-744’s BDII selectivity sets it apart from pan-BETi drugs like pelabresib and is considered to preserve therapeutic efficacy while lowering class-effect toxicity [135]. Thrombocytopenia (33%) and anemia (24%) were the most frequent TEAEs resulting in dose limiting toxicities, according to a Phase Ib study of ABBV-744 in MF patients who had previously undergone treatment. In terms of effectiveness, 33% of patients achieved spleen volume reduction by week 24 and 29% of patients had symptom control by week 12 [135].

Lysine-specific demethylase 1 (LSD1) modulates hematopoietic stem cell differentiation and has been recognized as a potential therapeutic target in hematological diseases [146]. LSD1 acts as a scaffold for the creation of chromatin-modifying complexes in addition to being a histone demethylase. Consequently, LSD1 performs both non-enzymatic and enzymatic functions [146]. LSD1 is irreversibly inhibited by a drug called Bomedemstat (MK-3543, IMG-7289). In a phase II trial, patients with ET who needed cytoreduction but did not respond to or were intolerant of ≥1 standard therapy were given bomedemstat. 77% of those receiving bomedemstat had normalized platelet levels and no new thromboembolic events. Additionally, it was considered safe and well-tolerated, with 86% of individuals continuing therapy after 24 weeks [136].

Immunotherapeutic targeting of mutant calreticulin (CALR) is a novel and promising approach in MPNs, as CALR mutations are associated with ET and primary MF, two major MPN subtypes. Exon 9 insertions or deletions are the most common type of CALR mutations [143]. Despite the fact that over 50 distinct exon 9 CALR mutations have been discovered, they all produce a neo-C-terminal sequence that is identical, leading to in a disease-specific neoantigen that is a compelling target for immunotherapy in MPNs [143]. A monoclonal antibody called INCA033989 specifically targets mutant CALR and prevents its carcinogenic activity without disrupting normal hematopoiesis. In vivo, INCA033989 effectively suppressed the growth of mutant CALR-expressing cells in mouse models, diminishing the number of mutant cells in the spleen and blood while maintaining normal hematopoiesis [147]. Currently, INCA033989 is being assessed in several clinical trials, including trials in healthy participants (NCT07448155), in participants with MPNs (NCT05936359), and in participants with ET (NCT07623200) [147].

Immunotherapies focused on T cells, including bispecific CD3 redirection antibodies, have been recognized as another promising treatment option. JNJ-88549968 is a new T-cell-redirecting bispecific antibody that preferentially targets mutant CALR and has the potential to cure MPN by eradicating clones [139]. JNJ-88549968 essentially acts as a bridge across mutant CALR-expressing cells as well as T cells, producing T-cell-mediated cytotoxicity both in vitro and in vivo. JNJ-88549968 is now being tested in a phase I clinical trial (NCT06150157) to investigate the safety, pharmacokinetics, and pharmacodynamics in MPN patients [139].

CALR-mutant cells are attractive targets for vaccine treatment because they express immunogenic tumor-specific antigens. A phase I trial assessed the safety and immunogenicity of CALRLong36 peptide immunization in MF patients. No objective clinical responses were seen, despite the fact that this vaccination was well tolerated and most individuals exhibited a noticeable T-cell response to it at the end of the trial [141].

### 6.3. Preclinical Approaches

In addition to clinical-stage investigational drugs, emerging preclinical approaches utilize novel biochemical mechanisms and targeted cell therapies to selectively eliminate malignant hematopoietic stem and progenitor cells.

A novel approach called proteolysis targeting chimera (PROTAC) targets a particular protein and causes its breakdown in the cell by using the ubiquitin–proteasome pathway. Numerous studies have demonstrated the advantages of protein degradation over protein inhibition for anticancer treatment strategies [148]. In preclinical MPN models, Compound 10i, a PROTAC degrader of JAK2, shows promising therapeutic potential. Compound 10i, which degrades JAK2 via the ubiquitin–proteasome pathway, has a greater antiproliferative effect in SET-2 cells relative to both fedratinib and its parent agent WWQ-131. Importantly, 10i suppresses recombinant human erythropoietin (rhEPO)-induced polycythemia and splenomegaly in murine models by depleting JAK2 protein levels and disrupting downstream JAK2-STAT signaling [132].

Chimeric antigen receptor (CAR) T-cell therapy has become a significant development in individualized cancer care. This method involves genetically modifying a patient’s own T cells to produce an engineered receptor capable of recognizing tumor-associated antigens [149]. For instance, CART3-mCALR is a novel CAR-T construct that demonstrated strong cytotoxicity against cell lines that expressed and did not express MPL. Additionally, CART3-mCALR cells successfully targeted MARIMO cells, the only known cell line naturally expressing mCALR, both in vitro and in vivo, despite their low antigen levels and absence of MPL expression. Furthermore, experiments utilizing a lower dose of MARIMO cells, which represent slower growth comparable to the early stages of the disease in patients, revealed a strong long-term efficacy of the CAR-T cells. All these findings point to CART3-mCALR cells as a promising therapeutic strategy in MPNs [143].

CALR × CDK9 represents a new precision antibody–drug conjugate (pADC) engineered to specifically engage mutant CALR and transport a targeted CDK9 degrader payload. In preclinical models, CALR-mutant cells exhibited substantial cytotoxicity and selective CDK9 degradation when treated with the CALR × CDK9 pADC [144].

Targeting the NLRP3 inflammasome is an emerging therapeutic strategy in MPNs, as supported by preclinical evidence showing that MCC950, a selective NLRP3 inhibitor, reduces IL-1β and IL-18 levels in inflammatory disease models [128].

### 6.4. Combination Therapies Targeting MPNs

Combination therapy has emerged as a pivotal approach to improving treatment outcomes and addressing the limitations of JAK inhibitor monotherapy. In the context of MPNs, combinations that pair FDA-approved JAK inhibitors with other emerging therapeutic agents are being explored to achieve enhanced efficacy through synergistic mechanisms of action.

Pelabresib (CPI-0610) is a small-molecule BET inhibitor under investigation in combination with ruxolitinib. The phase III MANIFEST-2 trial (NCT04603495) compared pelabresib plus ruxolitinib to placebo plus ruxolitinib in JAK inhibitor-naive individuals with MF and reported significant improvements in spleen volume reduction and symptom burden [150]. In comparison to 35.2% of patients receiving placebo plus ruxolitinib, 65.9% of patients receiving pelabresib plus ruxolitinib exhibited a reduction in spleen volume of ≥35%, surpassing response rates documented in earlier studies of JAK inhibitor monotherapy. The study found a significant decrease in proinflammatory cytokines such as NF-κB-regulated cytokines, TNF, IL-6, and IL-8 compared to placebo plus ruxolitinib [150]. The combination was well tolerated, with thrombocytopenia and anemia being the most frequent TEAEs. Importantly, hemoglobin concentrations increased, and the proportion of patients needing transfusions during the first 24 weeks of therapy was lower among those receiving pelabresib plus ruxolitinib compared to those getting placebo plus ruxolitinib (27.6% vs. 37.5%) [150].

As previously mentioned, vaccines targeting CALR mutant cells have an acceptable safety profile, but they failed to produce a meaningful therapeutic benefit since no patient’s disease status improved. As a result, these vaccines have been combined with other immunotherapeutic strategies to improve clinical efficacy [141]. VAC85135 is a peptide vaccine that contains epitopes derived from mutant CALR and JAK2V617F. A phase I trial evaluated VAC85135 in combination with ipilimumab, an anti-CTLA-4 checkpoint inhibitor [151]. Although this regimen was well tolerated, no evidence of disease-modifying efficacy was observed. The researchers concluded that the regimen did not warrant additional clinical development for the treatment of MPNs since the study revealed that VAC85135 was unable to yield a robust neoantigen-specific antitumor immune response [151].

## 7. Conclusions

Ph-negative myeloproliferative neoplasms are clonal hematopoietic diseases caused by mutations in JAK2, CALR, and MPL. These mutations promote activation of the JAK–STAT pathway and abnormally regulate NF-κB signaling, resulting in a chronic inflammatory condition. This article reviewed how the abnormal cytokine milieu, which is dominated by TGF-β, IL-13, TNF-α, IL-6, IL-8, and VEGF, contributes to clinical expressions of bone marrow fibrosis, splenomegaly, thrombosis, and leukemic transformation. Cooperating epigenetic mutations in TET2, DNMT3A, ASXL1, and EZH2 enhance inflammatory signals as well as accelerate clonal evolution. Progressive immune system dysfunction, including T and B cell exhaustion, NK cell maturation arrest, defective antigen presentation, MDSC accumulation, and abnormal monocyte and macrophage activity, all contribute to tumor immune escape as well as disease persistence. Neutrophil-driven NETosis and platelet-mediated cytokine amplification exacerbate the prothrombotic and pro-inflammatory environment. The FDA-approved JAK1/JAK2 inhibitors ruxolitinib, fedratinib, pacritinib, and momelotinib efficiently decrease splenomegaly as well as symptom burden, but have had a minor effect on progression of the disease and survival. Emerging therapies, such as pelabresib in combination with ruxolitinib, CALR-targeted monoclonal antibodies like INCA033989, bispecific T cell-redirecting antibodies, as well as mutation-specific peptide vaccines, show promise for long-term disease modification. In summary, these results highlight the importance of understanding the connections between driver mutations, inflammatory signaling, and immune dysregulation within the bone marrow niche in developing more efficient MPN therapies.

## Figures and Tables

**Table 1:** Cytokine, Chemokine, and Growth Factor Profiles in Myeloproliferative Neoplasm Subtypes.

MPN Subtype	Upregulated Cytokines, Chemokines, and Growth Factors	Refs.
Polycythemia Vera (PV)	VEGF, HGF, IL-11, IL-12, TGF-α, IL-1RA, IL-5, IL-6, IL-7, IL-8, IL-10, IL-13, IFN-α, IFN-γ, MCP-1, MIP-1α, MIP-1β, IP-10, MIG, IL-23, IL-22	[50,51,52]
Essential Thrombocythemia (ET)	GRO-α (CXCL1), EGF, PDGF, IL-4, IL-5, IL-6, IL-8, IL-10, IL-22, IFN-γ, MCP-1, VEGF	[50,53]
Primary Myelofibrosis (PMF)	TGF-β, IL-13, IL-8, IL-12, IL-15, IL-17 (IL-17A), TNF-α, IP-10, TIMP-1, IL-1β, IL-1RA, IL-2, IL-2R/sIL-2R, IL-4, IL-6, IL-10, HGF, VEGF, MIP-1α, MIP-1β, IL-13	[53,54]

**Table 2:** Biological Functions of Cytokines, Chemokines, and Growth Factors in Myeloproliferative Neoplasms, Grouped by Biological Relevance.

Functional Category	Representative Mediators	Major Biological/Clinical Relevance
Fibrosis-related	TGF-β, FGF, IL-11, IL-13, TPO	Fibrotic signaling, collagen synthesis, megakaryocyte expansion and bone-marrow remodeling; associated with MF progression [44,52,55,56,57,58,59,60]
Thrombo-inflammatory	IL-1/IL-1β, IL-6, IL-8, TNF-α, IFN-α, IFN-γ, CCL3, CCL4, CXCL9, CCL2, CXCL10, CXCL1	Inflammatory signaling, leukocytosis, myeloid-cell recruitment, neutrophil activation and thrombo-inflammatory responses [53,54,59,61,62,63,64,65,66,67,68,69]
Angiogenic	VEGF, HIF-1α, HGF	Bone-marrow angiogenesis, neovascularization and inflammation–angiogenesis interactions [4,36,57,70]
Immunoregulatory/immune-suppressive	IL-1RA, IL-4, IL-10	Modulation of inflammatory and immune responses [53,60,71]
Prognostic markers	EGF, IL-2, sIL-2R, IL-8, IL-12, CCL4, CXCL10, CXCL1	Associations with reduced survival, transfusion dependence, leukemic transformation or disease progression [53,54,59,63,64,68,69,72,73]

**Table 3:** Summary of Agents Currently Being Investigated.

Therapeutic Class	Agent	Main Mechanism of Action	Disease	Model	Ref./Identifier
Novel/Selective JAK Inhibitors	Rovadicitinib (TQ05105)	JAK/ROCK small-molecule inhibitor	MF	Phase II	[129] NCT07551427
	Itacitinib	JAK1 inhibitor	MF	Phase II	[130] NCT03144687
	PRT12396	mutant-selective JAK2V617F inhibitor	PV, MF	Phase I	NCT07469891
	AJ1-11095	type-II JAK2 inhibitor	PMF, PPV-MF, PET-MF	Phase I	NCT06343805 [131]
	Compound 10i	PROTAC JAK2 inhibitor	MPNs	Preclinical	[132]
	WWQ-131	small molecule JAK2 inhibitor	MPNs	Preclinical	[133]
Epigenetic Modulators	Pelabresib (CPI-0610)	small molecule BET inhibitor	MF	Phase III	[134] NCT04603495
	ABBV-744	small molecule BET inhibitor	MF	Phase Ib	[135] NCT04454658
	Bomedemstat (IMG-7289)	LSD1 irreversible inhibitor	ET	Phase II	[136] NCT04254978
	Givinostat	HDAC inhibitor	PV	Phase III	NCT06093672
	Vorinostat	HDAC inhibitor	MF	Phase II	[137]
CALR-Directed Immunotherapies	INCA033989	monoclonal antibody selectively targeting CALR mutant cells	ET, MF	Phase I	[138] NCT05936359 NCT06034002
	JNJ-88549968	bispecific Antibody selectively inhibitor of CALR mutant cells	ET, MF	Phase I	[139] NCT06150157
	INCA035784	bispecific Antibody selectively inhibitor of CALR mutant cells	ET, PMF	Phase I	[140] NCT07008118
	CALRLong36 peptide	Peptide vaccine against CALR mutant cells	ET, MF	Phase I	[141] NCT03566446
	Mutant-CALR Peptide Vaccine	Peptide vaccine against CALR mutant cells	ET, MF	Phase I	[142] NCT05025488
	VAC85135	Peptide vaccine against CALR mutant cells	MPNs	Phase I	NCT05444530
	CART3-mCALR	CAR T-cell therapy	MPNs	Preclinical	[143]
	CALR × CDK9 pADC	CALR-targeted precision antibody–drug conjugate (ADC)	MPNs	Preclinical	[144]

## Data Availability

No new data were created or analyzed in this study. Data sharing is not applicable to this article.
